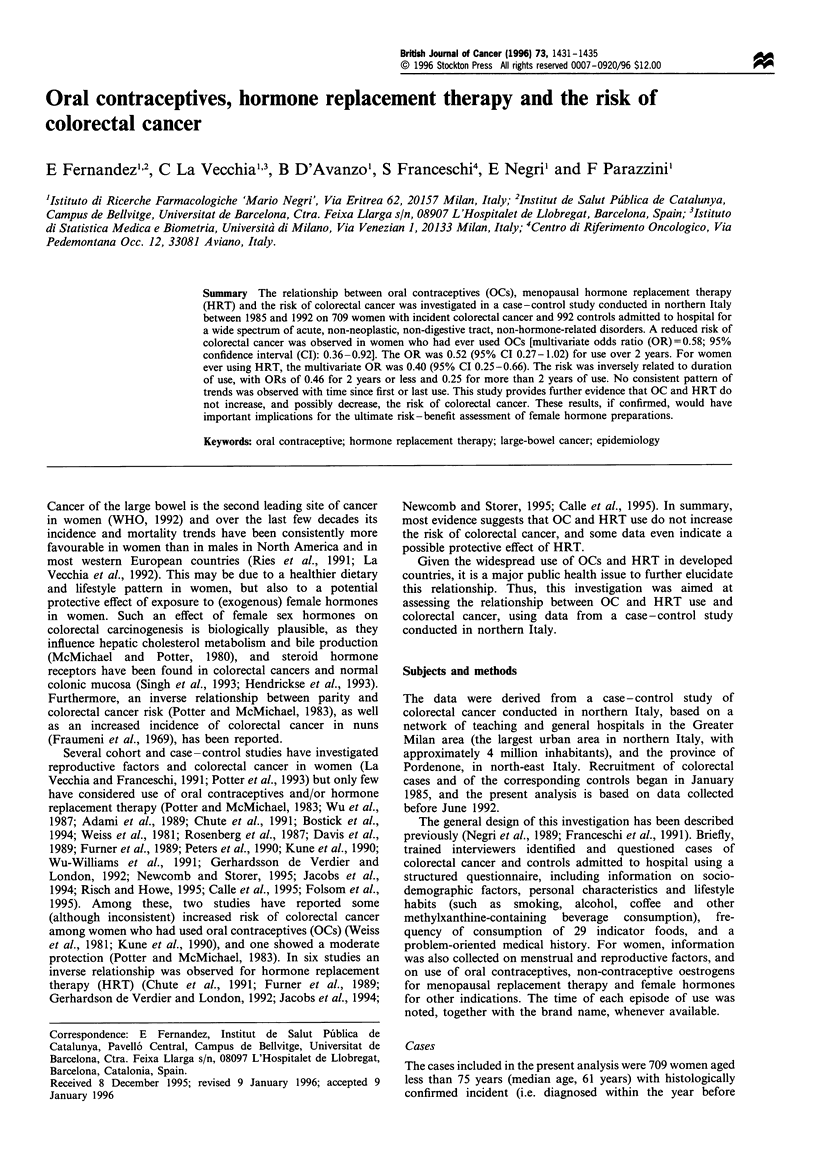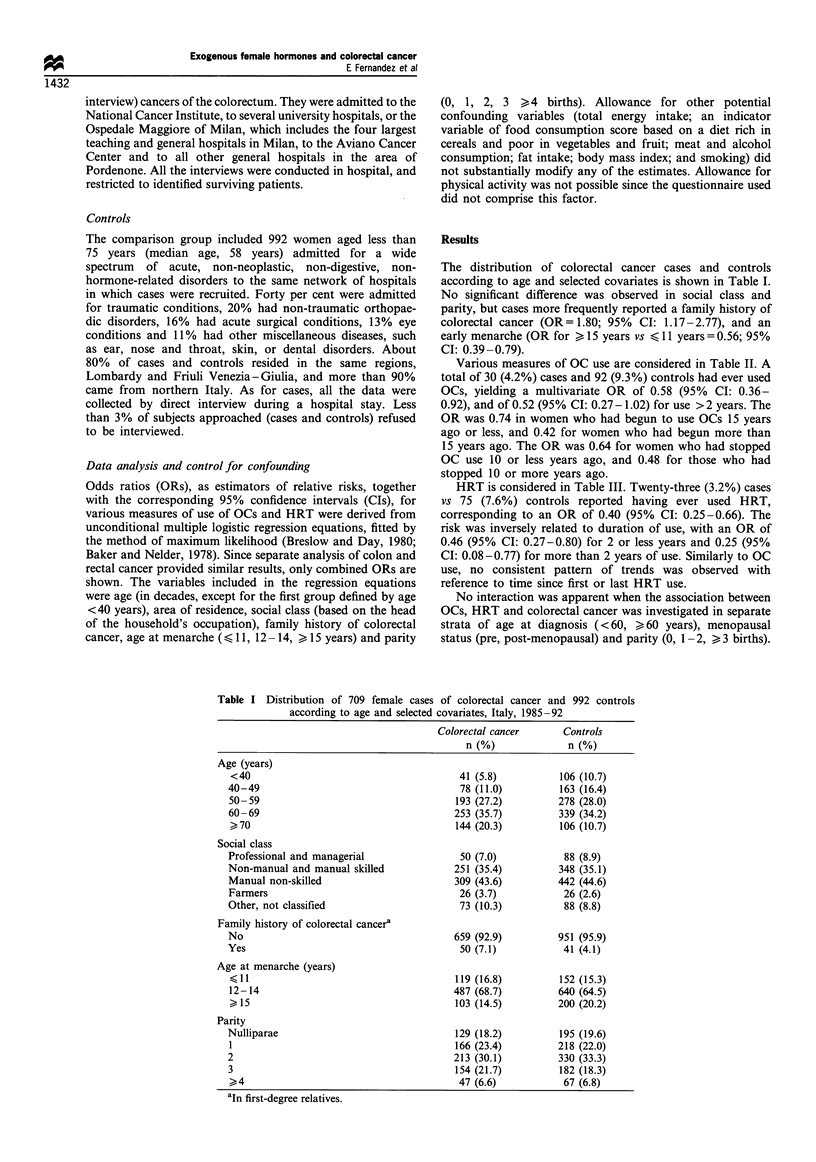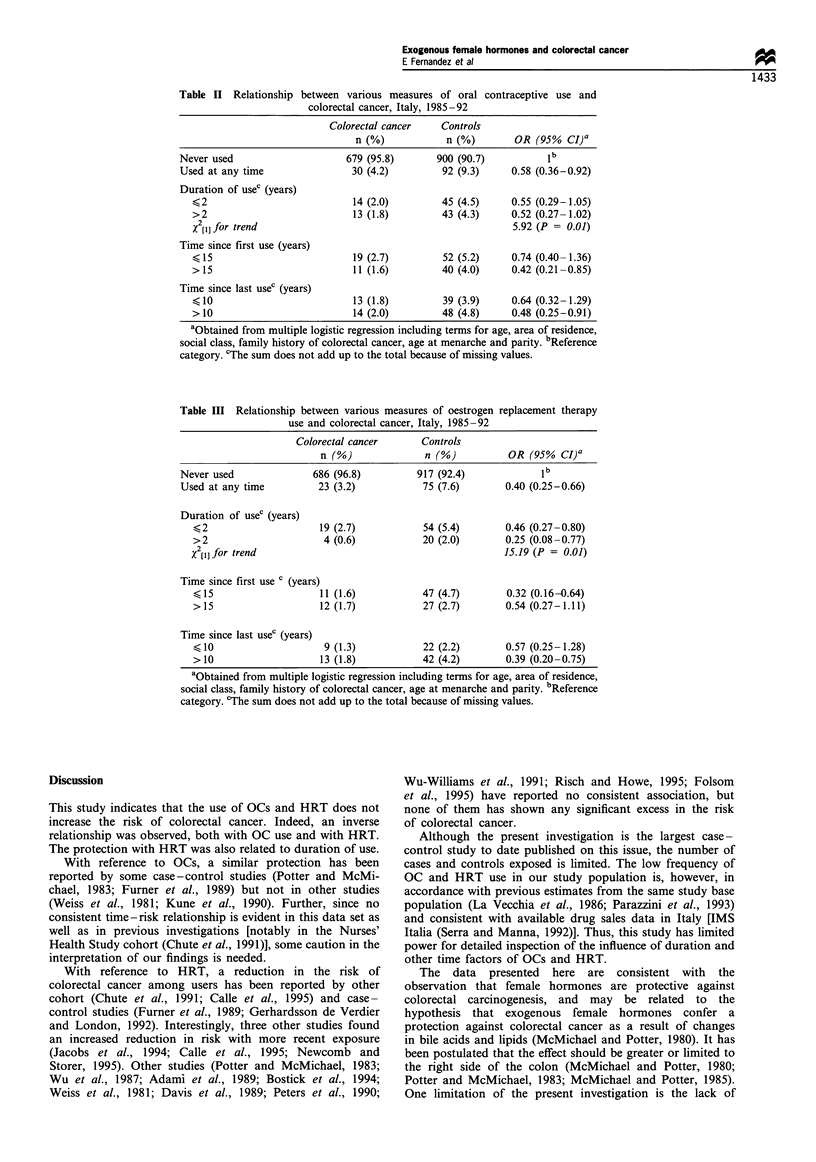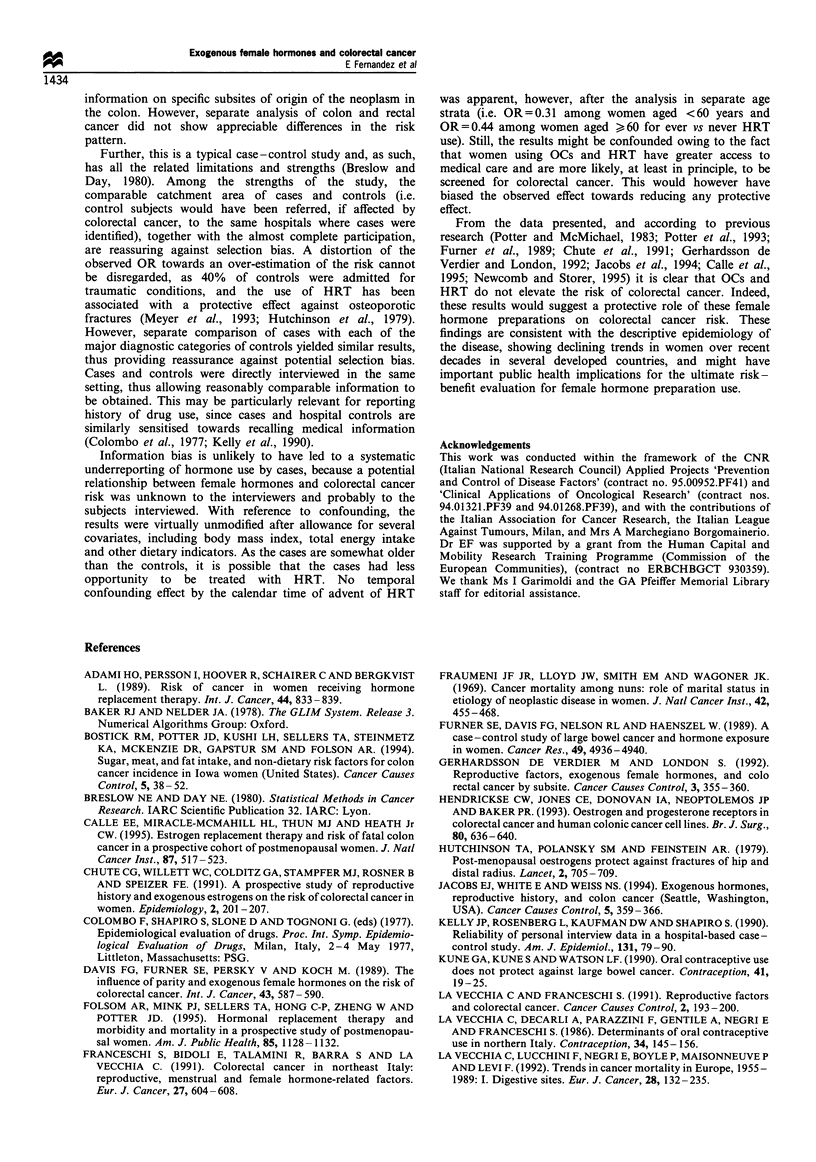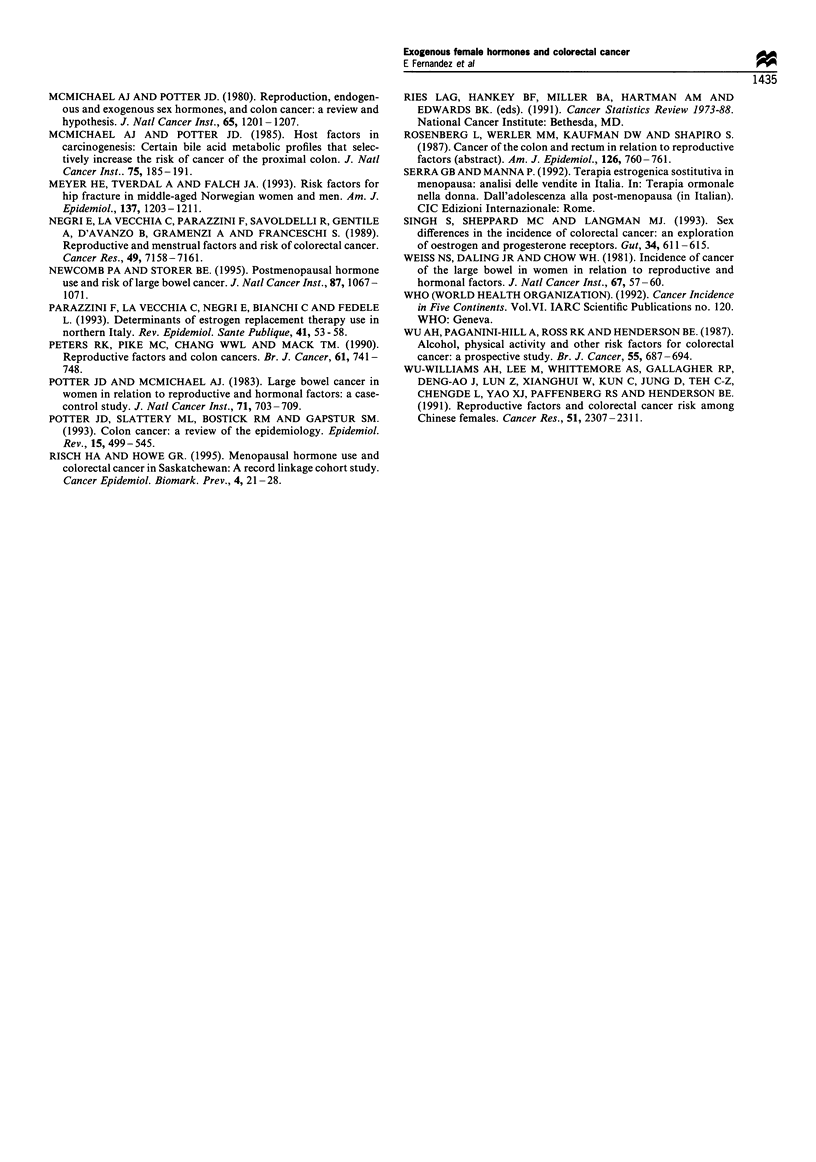# Oral contraceptives, hormone replacement therapy and the risk of colorectal cancer.

**DOI:** 10.1038/bjc.1996.272

**Published:** 1996-06

**Authors:** E. Fernandez, C. La Vecchia, B. D'Avanzo, S. Franceschi, E. Negri, F. Parazzini

**Affiliations:** Istituto di Ricerche Farmacologiche Mario Negri, Milan, Italy.

## Abstract

The relationship between oral contraceptives (OCs), menopausal hormone replacement therapy (HRT) and the risk of colorectal cancer was investigated in a case-control study conducted in northern Italy between 1985 and 1992 on 709 women with incident colorectal cancer and 992 controls admitted to hospital for a wide spectrum of acute, non-neoplastic, non-digestive tract, non-hormone-related disorders. A reduced risk of colorectal cancer was observed in women who had ever used OCs [multivariate odds ratio (OR) = 0.58; 95% confidence interval (CI): 0.36-0.92]. The OR was 0.52 (95% CI 0.27-1.02) for use over 2 years. For women ever using HRT, the multivariate OR was 0.40 (95% CI 0.25-0.66). The risk was inversely related to duration of use, with ORs of 0.46 for 2 years or less and 0.25 for more than 2 years of use. No consistent pattern of trends was observed with time since first or last use. This study provides further evidence that OC and HRT do not increase, and possibly decrease, the risk of colorectal cancer. These results, if confirmed, would have important implications for the ultimate risk-benefit assessment of female hormone preparations.


British Journal of Cancer (1996) 73, 1431-1435
© 1996 Stockton Press All rights reserved 0007-0920/96 $12.00

### Oral contraceptives, hormone replacement therapy and the risk of colorectal cancer

E Fernandez[1,2], C La Vecchia[1,3], B D'Avanzo[1], S Franceschi[4], E Negri[1] and F Parazzini[1]

*[1]Istituto di Ricerche Farmacologiche 'Mario Negri', Via Eritrea 62, 20157 Milan, Italy; [2]Institut de Salut Pública de Catalunya, Campus de Bellvitge, Universitat de Barcelona, Ctra. Feixa Llarga s/n, 08907 L'Hospitalet de Llobregat, Barcelona, Spain; [3]Istituto di Statistica Medica e Biometria, Università di Milano, Via Venezian 1, 20133 Milan, Italy; [4]Centro di Riferimento Oncologico, Via Pedemontana Occ. 12, 33081 Aviano, Italy.*

**Summary** The relationship between oral contraceptives (OCs), menopausal hormone replacement therapy (HRT) and the risk of colorectal cancer was investigated in a case-control study conducted in northern Italy between 1985 and 1992 on 709 women with incident colorectal cancer and 992 controls admitted to hospital for a wide spectrum of acute, non-neoplastic, non-digestive tract, non-hormone-related disorders. A reduced risk of colorectal cancer was observed in women who had ever used OCs [multivariate odds ratio (OR)=0.58; 95% confidence interval (CI): 0.36-0.92]. The OR was 0.52 (95% CI 0.27-1.02) for use over 2 years. For women ever using HRT, the multivariate OR was 0.40 (95% CI 0.25-0.66). The risk was inversely related to duration of use, with ORs of 0.46 for 2 years or less and 0.25 for more than 2 years of use. No consistent pattern of trends was observed with time since first or last use. This study provides further evidence that OC and HRT do not increase, and possibly decrease, the risk of colorectal cancer. These results, if confirmed, would have important implications for the ultimate risk-benefit assessment of female hormone preparations.

**Keywords:** oral contraceptive; hormone replacement therapy; large-bowel cancer; epidemiology

Cancer of the large bowel is the second leading site of cancer in women (WHO, 1992) and over the last few decades its incidence and mortality trends have been consistently more favourable in women than in males in North America and in most western European countries (Ries *et al.*, 1991; La Vecchia *et al.*, 1992). This may be due to a healthier dietary and lifestyle pattern in women, but also to a potential protective effect of exposure to (exogenous) female hormones in women. Such an effect of female sex hormones on colorectal carcinogenesis is biologically plausible, as they influence hepatic cholesterol metabolism and bile production (McMichael and Potter, 1980), and steroid hormone receptors have been found in colorectal cancers and normal colonic mucosa (Singh *et al.*, 1993; Hendrickse *et al.*, 1993). Furthermore, an inverse relationship between parity and colorectal cancer risk (Potter and McMichael, 1983), as well as an increased incidence of colorectal cancer in nuns (Fraumeni *et al.*, 1969), has been reported.

Several cohort and case-control studies have investigated reproductive factors and colorectal cancer in women (La Vecchia and Franceschi, 1991; Potter *et al.*, 1993) but only few have considered use of oral contraceptives and/or hormone replacement therapy (Potter and McMichael, 1983; Wu *et al.*, 1987; Adami *et al.*, 1989; Chute *et al.*, 1991; Bostick *et al.*, 1994; Weiss *et al.*, 1981; Rosenberg *et al.*, 1987; Davis *et al.*, 1989; Furner *et al.*, 1989; Peters *et al.*, 1990; Kune *et al.*, 1990; Wu-Williams *et al.*, 1991; Gerhardsson de Verdier and London, 1992; Newcomb and Storer, 1995; Jacobs *et al.*, 1994; Risch and Howe, 1995; Calle *et al.*, 1995; Folsom *et al.*, 1995). Among these, two studies have reported some (although inconsistent) increased risk of colorectal cancer among women who had used oral contraceptives (OCs) (Weiss *et al.*, 1981; Kune *et al.*, 1990), and one showed a moderate protection (Potter and McMichael, 1983). In six studies an inverse relationship was observed for hormone replacement therapy (HRT) (Chute *et al.*, 1991; Furner *et al.*, 1989; Gerhardson de Verdier and London, 1992; Jacobs *et al.*, 1994; Newcomb and Storer, 1995; Calle *et al.*, 1995). In summary, most evidence suggests that OC and HRT use do not increase the risk of colorectal cancer, and some data even indicate a possible protective effect of HRT.

Given the widespread use of OCs and HRT in developed countries, it is a major public health issue to further elucidate this relationship. Thus, this investigation was aimed at assessing the relationship between OC and HRT use and colorectal cancer, using data from a case-control study conducted in northern Italy.

### Subjects and methods

The data were derived from a case-control study of colorectal cancer conducted in northern Italy, based on a network of teaching and general hospitals in the Greater Milan area (the largest urban area in northern Italy, with approximately 4 million inhabitants), and the province of Pordenone, in north-east Italy. Recruitment of colorectal cases and of the corresponding controls began in January 1985, and the present analysis is based on data collected before June 1992.

The general design of this investigation has been described previously (Negri *et al.*, 1989; Franceschi *et al.*, 1991). Briefly, trained interviewers identified and questioned cases of colorectal cancer and controls admitted to hospital using a structured questionnaire, including information on socio-demographic factors, personal characteristics and lifestyle habits (such as smoking, alcohol, coffee and other methylxanthine-containing beverage consumption), frequency of consumption of 29 indicator foods, and a problem-oriented medical history. For women, information was also collected on menstrual and reproductive factors, and on use of oral contraceptives, non-contraceptive oestrogens for menopausal replacement therapy and female hormones for other indications. The time of each episode of use was noted, together with the brand name, whenever available.

### *Cases*

The cases included in the present analysis were 709 women aged less than 75 years (median age, 61 years) with histologically confirmed incident (i.e. diagnosed within the year before

Correspondence: E Fernandez, Institut de Salut Pública de Catalunya, Pavelló Central, Campus de Bellvitge, Universitat de Barcelona, Ctra. Feixa Llarga s/n, 08097 L'Hospitalet de Llobregat, Barcelona, Catalonia, Spain.
Received 8 December 1995; revised 9 January 1996; accepted 9 January 1996

interview) cancers of the colorectum. They were admitted to the National Cancer Institute, to several university hospitals, or the Ospedale Maggiore of Milan, which includes the four largest teaching and general hospitals in Milan, to the Aviano Cancer Center and to all other general hospitals in the area of Pordenone. All the interviews were conducted in hospital, and restricted to identified surviving patients.

### *Controls*

The comparison group included 992 women aged less than 75 years (median age, 58 years) admitted for a wide spectrum of acute, non-neoplastic, non-digestive, non-hormone-related disorders to the same network of hospitals in which cases were recruited. Forty per cent were admitted for traumatic conditions, 20% had non-traumatic orthopaedic disorders, 16% had acute surgical conditions, 13% eye conditions and 11% had other miscellaneous diseases, such as ear, nose and throat, skin, or dental disorders. About 80% of cases and controls resided in the same regions, Lombardy and Friuli Venezia–Giulia, and more than 90% came from northern Italy. As for cases, all the data were collected by direct interview during a hospital stay. Less than 3% of subjects approached (cases and controls) refused to be interviewed.

### *Data analysis and control for confounding*

Odds ratios (ORs), as estimators of relative risks, together with the corresponding 95% confidence intervals (CIs), for various measures of use of OCs and HRT were derived from unconditional multiple logistic regression equations, fitted by the method of maximum likelihood (Breslow and Day, 1980; Baker and Nelder, 1978). Since separate analysis of colon and rectal cancer provided similar results, only combined ORs are shown. The variables included in the regression equations were age (in decades, except for the first group defined by age <40 years), area of residence, social class (based on the head of the household's occupation), family history of colorectal cancer, age at menarche (⩽11, 12–14, ⩾15 years) and parity (0, 1, 2, 3 ⩾4 births). Allowance for other potential confounding variables (total energy intake; an indicator variable of food consumption score based on a diet rich in cereals and poor in vegetables and fruit; meat and alcohol consumption; fat intake; body mass index; and smoking) did not substantially modify any of the estimates. Allowance for physical activity was not possible since the questionnaire used did not comprise this factor.

### Results

The distribution of colorectal cancer cases and controls according to age and selected covariates is shown in Table I. No significant difference was observed in social class and parity, but cases more frequently reported a family history of colorectal cancer (OR = 1.80; 95% CI: 1.17–2.77), and an early menarche (OR for ⩾15 years *vs* ⩽11 years = 0.56; 95% CI: 0.39–0.79).

Various measures of OC use are considered in Table II. A total of 30 (4.2%) cases and 92 (9.3%) controls had ever used OCs, yielding a multivariate OR of 0.58 (95% CI: 0.36–0.92), and of 0.52 (95% CI: 0.27–1.02) for use >2 years. The OR was 0.74 in women who had begun to use OCs 15 years ago or less, and 0.42 for women who had begun more than 15 years ago. The OR was 0.64 for women who had stopped OC use 10 or less years ago, and 0.48 for those who had stopped 10 or more years ago.

HRT is considered in Table III. Twenty-three (3.2%) cases *vs* 75 (7.6%) controls reported having ever used HRT, corresponding to an OR of 0.40 (95% CI: 0.25–0.66). The risk was inversely related to duration of use, with an OR of 0.46 (95% CI: 0.27–0.80) for 2 or less years and 0.25 (95% CI: 0.08–0.77) for more than 2 years of use. Similarly to OC use, no consistent pattern of trends was observed with reference to time since first or last HRT use.

No interaction was apparent when the association between OCs, HRT and colorectal cancer was investigated in separate strata of age at diagnosis (<60, ⩾60 years), menopausal status (pre, post-menopausal) and parity (0, 1–2, ⩾3 births).

**Table I** Distribution of 709 female cases of colorectal cancer and 992 controls according to age and selected covariates, Italy, 1985–92

| | *Colorectal cancer* n (%) | *Controls* n (%) |
|---|---|---|
| Age (years) | | |
| <40 | 41 (5.8) | 106 (10.7) |
| 40–49 | 78 (11.0) | 163 (16.4) |
| 50–59 | 193 (27.2) | 278 (28.0) |
| 60–69 | 253 (35.7) | 339 (34.2) |
| ⩾70 | 144 (20.3) | 106 (10.7) |
| Social class | | |
| Professional and managerial | 50 (7.0) | 88 (8.9) |
| Non-manual and manual skilled | 251 (35.4) | 348 (35.1) |
| Manual non-skilled | 309 (43.6) | 442 (44.6) |
| Farmers | 26 (3.7) | 26 (2.6) |
| Other, not classified | 73 (10.3) | 88 (8.8) |
| Family history of colorectal cancer[a] | | |
| No | 659 (92.9) | 951 (95.9) |
| Yes | 50 (7.1) | 41 (4.1) |
| Age at menarche (years) | | |
| ⩽11 | 119 (16.8) | 152 (15.3) |
| 12–14 | 487 (68.7) | 640 (64.5) |
| ⩾15 | 103 (14.5) | 200 (20.2) |
| Parity | | |
| Nulliparae | 129 (18.2) | 195 (19.6) |
| 1 | 166 (23.4) | 218 (22.0) |
| 2 | 213 (30.1) | 330 (33.3) |
| 3 | 154 (21.7) | 182 (18.3) |
| ⩾4 | 47 (6.6) | 67 (6.8) |

[a]In first-degree relatives.

**Table II** Relationship between various measures of oral contraceptive use and colorectal cancer, Italy, 1985–92

| | *Colorectal cancer* n (%) | *Controls* n (%) | *OR (95% CI)*[a] |
|---|---|---|---|
| Never used | 679 (95.8) | 900 (90.7) | 1[b] |
| Used at any time | 30 (4.2) | 92 (9.3) | 0.58 (0.36–0.92) |
| Duration of use[c] (years) | | | |
| ⩽2 | 14 (2.0) | 45 (4.5) | 0.55 (0.29–1.05) |
| >2 | 13 (1.8) | 43 (4.3) | 0.52 (0.27–1.02) |
| $\chi^2_{[1]}$ *for trend* | | | 5.92 (*P = 0.01*) |
| Time since first use (years) | | | |
| ⩽15 | 19 (2.7) | 52 (5.2) | 0.74 (0.40–1.36) |
| >15 | 11 (1.6) | 40 (4.0) | 0.42 (0.21–0.85) |
| Time since last use[c] (years) | | | |
| ⩽10 | 13 (1.8) | 39 (3.9) | 0.64 (0.32–1.29) |
| >10 | 14 (2.0) | 48 (4.8) | 0.48 (0.25–0.91) |

[a]Obtained from multiple logistic regression including terms for age, area of residence, social class, family history of colorectal cancer, age at menarche and parity. [b]Reference category. [c]The sum does not add up to the total because of missing values.

**Table III** Relationship between various measures of oestrogen replacement therapy use and colorectal cancer, Italy, 1985–92

| | *Colorectal cancer* n *(%)* | *Controls* *n (%)* | *OR (95% CI)*[a] |
|---|---|---|---|
| Never used | 686 (96.8) | 917 (92.4) | 1[b] |
| Used at any time | 23 (3.2) | 75 (7.6) | 0.40 (0.25–0.66) |
| Duration of use[c] (years) | | | |
| ⩽2 | 19 (2.7) | 54 (5.4) | 0.46 (0.27–0.80) |
| >2 | 4 (0.6) | 20 (2.0) | 0.25 (0.08–0.77) |
| $\chi^2_{[1]}$ *for trend* | | | *15.19* (*P = 0.01*) |
| Time since first use [c] (years) | | | |
| ⩽15 | 11 (1.6) | 47 (4.7) | 0.32 (0.16–0.64) |
| >15 | 12 (1.7) | 27 (2.7) | 0.54 (0.27–1.11) |
| Time since last use[c] (years) | | | |
| ⩽10 | 9 (1.3) | 22 (2.2) | 0.57 (0.25–1.28) |
| >10 | 13 (1.8) | 42 (4.2) | 0.39 (0.20–0.75) |

[a]Obtained from multiple logistic regression including terms for age, area of residence, social class, family history of colorectal cancer, age at menarche and parity. [b]Reference category. [c]The sum does not add up to the total because of missing values.

### Discussion

This study indicates that the use of OCs and HRT does not increase the risk of colorectal cancer. Indeed, an inverse relationship was observed, both with OC use and with HRT. The protection with HRT was also related to duration of use.

With reference to OCs, a similar protection has been reported by some case–control studies (Potter and McMichael, 1983; Furner *et al.*, 1989) but not in other studies (Weiss *et al.*, 1981; Kune *et al.*, 1990). Further, since no consistent time–risk relationship is evident in this data set as well as in previous investigations [notably in the Nurses' Health Study cohort (Chute *et al.*, 1991)], some caution in the interpretation of our findings is needed.

With reference to HRT, a reduction in the risk of colorectal cancer among users has been reported by other cohort (Chute *et al.*, 1991; Calle *et al.*, 1995) and case–control studies (Furner *et al.*, 1989; Gerhardsson de Verdier and London, 1992). Interestingly, three other studies found an increased reduction in risk with more recent exposure (Jacobs *et al.*, 1994; Calle *et al.*, 1995; Newcomb and Storer, 1995). Other studies (Potter and McMichael, 1983; Wu *et al.*, 1987; Adami *et al.*, 1989; Bostick *et al.*, 1994; Weiss *et al.*, 1981; Davis *et al.*, 1989; Peters *et al.*, 1990; Wu-Williams *et al.*, 1991; Risch and Howe, 1995; Folsom *et al.*, 1995) have reported no consistent association, but none of them has shown any significant excess in the risk of colorectal cancer.

Although the present investigation is the largest case–control study to date published on this issue, the number of cases and controls exposed is limited. The low frequency of OC and HRT use in our study population is, however, in accordance with previous estimates from the same study base population (La Vecchia *et al.*, 1986; Parazzini *et al.*, 1993) and consistent with available drug sales data in Italy [IMS Italia (Serra and Manna, 1992)]. Thus, this study has limited power for detailed inspection of the influence of duration and other time factors of OCs and HRT.

The data presented here are consistent with the observation that female hormones are protective against colorectal carcinogenesis, and may be related to the hypothesis that exogenous female hormones confer a protection against colorectal cancer as a result of changes in bile acids and lipids (McMichael and Potter, 1980). It has been postulated that the effect should be greater or limited to the right side of the colon (McMichael and Potter, 1980; Potter and McMichael, 1983; McMichael and Potter, 1985). One limitation of the present investigation is the lack of information on specific subsites of origin of the neoplasm in the colon. However, separate analysis of colon and rectal cancer did not show appreciable differences in the risk pattern.

Further, this is a typical case–control study and, as such, has all the related limitations and strengths (Breslow and Day, 1980). Among the strengths of the study, the comparable catchment area of cases and controls (i.e. control subjects would have been referred, if affected by colorectal cancer, to the same hospitals where cases were identified), together with the almost complete participation, are reassuring against selection bias. A distortion of the observed OR towards an over-estimation of the risk cannot be disregarded, as 40% of controls were admitted for traumatic conditions, and the use of HRT has been associated with a protective effect against osteoporotic fractures (Meyer *et al*., 1993; Hutchinson *et al*., 1979). However, separate comparison of cases with each of the major diagnostic categories of controls yielded similar results, thus providing reassurance against potential selection bias. Cases and controls were directly interviewed in the same setting, thus allowing reasonably comparable information to be obtained. This may be particularly relevant for reporting history of drug use, since cases and hospital controls are similarly sensitised towards recalling medical information (Colombo *et al*., 1977; Kelly *et al*., 1990).

Information bias is unlikely to have led to a systematic underreporting of hormone use by cases, because a potential relationship between female hormones and colorectal cancer risk was unknown to the interviewers and probably to the subjects interviewed. With reference to confounding, the results were virtually unmodified after allowance for several covariates, including body mass index, total energy intake and other dietary indicators. As the cases are somewhat older than the controls, it is possible that the cases had less opportunity to be treated with HRT. No temporal confounding effect by the calendar time of advent of HRT was apparent, however, after the analysis in separate age strata (i.e. OR = 0.31 among women aged $<60$ years and OR = 0.44 among women aged $\geqslant 60$ for ever *vs* never HRT use). Still, the results might be confounded owing to the fact that women using OCs and HRT have greater access to medical care and are more likely, at least in principle, to be screened for colorectal cancer. This would however have biased the observed effect towards reducing any protective effect.

From the data presented, and according to previous research (Potter and McMichael, 1983; Potter *et al*., 1993; Furner *et al*., 1989; Chute *et al*., 1991; Gerhardsson de Verdier and London, 1992; Jacobs *et al*., 1994; Calle *et al*., 1995; Newcomb and Storer, 1995) it is clear that OCs and HRT do not elevate the risk of colorectal cancer. Indeed, these results would suggest a protective role of these female hormone preparations on colorectal cancer risk. These findings are consistent with the descriptive epidemiology of the disease, showing declining trends in women over recent decades in several developed countries, and might have important public health implications for the ultimate risk–benefit evaluation for female hormone preparation use.

**Acknowledgements**

This work was conducted within the framework of the CNR (Italian National Research Council) Applied Projects 'Prevention and Control of Disease Factors' (contract no. 95.00952.PF41) and 'Clinical Applications of Oncological Research' (contract nos. 94.01321.PF39 and 94.01268.PF39), and with the contributions of the Italian Association for Cancer Research, the Italian League Against Tumours, Milan, and Mrs A Marchegiano Borgomainerio. Dr EF was supported by a grant from the Human Capital and Mobility Research Training Programme (Commission of the European Communities), (contract no ERBCHBGCT 930359). We thank Ms I Garimoldi and the GA Pfeiffer Memorial Library staff for editorial assistance.

### References

ADAMI HO, PERSSON I, HOOVER R, SCHAIRER C AND BERGKVIST L. (1989). Risk of cancer in women receiving hormone replacement therapy. *Int. J. Cancer*, **44**, 833–839.

BAKER RJ AND NELDER JA. (1978). *The GLIM System. Release 3*. Numerical Algorithms Group: Oxford.

BOSTICK RM, POTTER JD, KUSHI LH, SELLERS TA, STEINMETZ KA, MCKENZIE DR, GAPSTUR SM AND FOLSON AR. (1994). Sugar, meat, and fat intake, and non-dietary risk factors for colon cancer incidence in Iowa women (United States). *Cancer Causes Control*, **5**, 38–52.

BRESLOW NE AND DAY NE. (1980). *Statistical Methods in Cancer Research*. IARC Scientific Publication 32. IARC: Lyon.

CALLE EE, MIRACLE-MCMAHILL HL, THUN MJ AND HEATH Jr CW. (1995). Estrogen replacement therapy and risk of fatal colon cancer in a prospective cohort of postmenopausal women. *J. Natl Cancer Inst*., **87**, 517–523.

CHUTE CG, WILLETT WC, COLDITZ GA, STAMPFER MJ, ROSNER B AND SPEIZER FE. (1991). A prospective study of reproductive history and exogenous estrogens on the risk of colorectal cancer in women. *Epidemiology*, **2**, 201–207.

COLOMBO F, SHAPIRO S, SLONE D AND TOGNONI G. (eds) (1977). Epidemiological evaluation of drugs. *Proc. Int. Symp. Epidemiological Evaluation of Drugs*, Milan, Italy, 2–4 May 1977, Littleton, Massachusetts: PSG.

DAVIS FG, FURNER SE, PERSKY V AND KOCH M. (1989). The influence of parity and exogenous female hormones on the risk of colorectal cancer. *Int. J. Cancer*, **43**, 587–590.

FOLSOM AR, MINK PJ, SELLERS TA, HONG C-P, ZHENG W AND POTTER JD. (1995). Hormonal replacement therapy and morbidity and mortality in a prospective study of postmenopausal women. *Am. J. Public Health*, **85**, 1128–1132.

FRANCESCHI S, BIDOLI E, TALAMINI R, BARRA S AND LA VECCHIA C. (1991). Colorectal cancer in northeast Italy: reproductive, menstrual and female hormone-related factors. *Eur. J. Cancer*, **27**, 604–608.

FRAUMENI JF JR, LLOYD JW, SMITH EM AND WAGONER JK. (1969). Cancer mortality among nuns: role of marital status in etiology of neoplastic disease in women. *J. Natl Cancer Inst*., **42**, 455–468.

FURNER SE, DAVIS FG, NELSON RL AND HAENSZEL W. (1989). A case–control study of large bowel cancer and hormone exposure in women. *Cancer Res*., **49**, 4936–4940.

GERHARDSSON DE VERDIER M AND LONDON S. (1992). Reproductive factors, exogenous female hormones, and colo rectal cancer by subsite. *Cancer Causes Control*, **3**, 355–360.

HENDRICKSE CW, JONES CE, DONOVAN IA, NEOPTOLEMOS JP AND BAKER PR. (1993). Oestrogen and progesterone receptors in colorectal cancer and human colonic cancer cell lines. *Br. J. Surg*., **80**, 636–640.

HUTCHINSON TA, POLANSKY SM AND FEINSTEIN AR. (1979). Post-menopausal oestrogens protect against fractures of hip and distal radius. *Lancet*, **2**, 705–709.

JACOBS EJ, WHITE E AND WEISS NS. (1994). Exogenous hormones, reproductive history, and colon cancer (Seattle, Washington, USA). *Cancer Causes Control*, **5**, 359–366.

KELLY JP, ROSENBERG L, KAUFMAN DW AND SHAPIRO S. (1990). Reliability of personal interview data in a hospital-based case–control study. *Am. J. Epidemiol*., **131**, 79–90.

KUNE GA, KUNE S AND WATSON LF. (1990). Oral contraceptive use does not protect against large bowel cancer. *Contraception*, **41**, 19–25.

LA VECCHIA C AND FRANCESCHI S. (1991). Reproductive factors and colorectal cancer. *Cancer Causes Control*, **2**, 193–200.

LA VECCHIA C, DECARLI A, PARAZZINI F, GENTILE A, NEGRI E AND FRANCESCHI S. (1986). Determinants of oral contraceptive use in northern Italy. *Contraception*, **34**, 145–156.

LA VECCHIA C, LUCCHINI F, NEGRI E, BOYLE P, MAISONNEUVE P AND LEVI F. (1992). Trends in cancer mortality in Europe, 1955–1989: I. Digestive sites. *Eur. J. Cancer*, **28**, 132–235.

MCMICHAEL AJ AND POTTER JD. (1980). Reproduction, endogenous and exogenous sex hormones, and colon cancer: a review and hypothesis. *J. Natl Cancer Inst.*, **65**, 1201–1207.

MCMICHAEL AJ AND POTTER JD. (1985). Host factors in carcinogenesis: Certain bile acid metabolic profiles that selectively increase the risk of cancer of the proximal colon. *J. Natl Cancer Inst.*. **75**, 185–191.

MEYER HE, TVERDAL A AND FALCH JA. (1993). Risk factors for hip fracture in middle-aged Norwegian women and men. *Am. J. Epidemiol.*, **137**, 1203–1211.

NEGRI E, LA VECCHIA C, PARAZZINI F, SAVOLDELLI R, GENTILE A, D'AVANZO B, GRAMENZI A AND FRANCESCHI S. (1989). Reproductive and menstrual factors and risk of colorectal cancer. *Cancer Res.*, **49**, 7158–7161.

NEWCOMB PA AND STORER BE. (1995). Postmenopausal hormone use and risk of large bowel cancer. *J. Natl Cancer Inst.*, **87**, 1067–1071.

PARAZZINI F, LA VECCHIA C, NEGRI E, BIANCHI C AND FEDELE L. (1993). Determinants of estrogen replacement therapy use in northern Italy. *Rev. Epidemiol. Sante Publique*, **41**, 53-58.

PETERS RK, PIKE MC, CHANG WWL AND MACK TM. (1990). Reproductive factors and colon cancers. *Br. J. Cancer*, **61**, 741–748.

POTTER JD AND MCMICHAEL AJ. (1983). Large bowel cancer in women in relation to reproductive and hormonal factors: a case-control study. *J. Natl Cancer Inst.*, **71**, 703–709.

POTTER JD, SLATTERY ML, BOSTICK RM AND GAPSTUR SM. (1993). Colon cancer: a review of the epidemiology. *Epidemiol. Rev.*, **15**, 499–545.

RISCH HA AND HOWE GR. (1995). Menopausal hormone use and colorectal cancer in Saskatchewan: A record linkage cohort study. *Cancer Epidemiol. Biomark. Prev.*, **4**, 21–28.

RIES LAG, HANKEY BF, MILLER BA, HARTMAN AM AND EDWARDS BK. (eds). (1991). *Cancer Statistics Review 1973-88*. National Cancer Institute: Bethesda, MD.

ROSENBERG L, WERLER MM, KAUFMAN DW AND SHAPIRO S. (1987). Cancer of the colon and rectum in relation to reproductive factors (abstract). *Am. J. Epidemiol.*, **126**, 760–761.

SERRA GB AND MANNA P. (1992). Terapia estrogenica sostitutiva in menopausa: analisi delle vendite in Italia. In: Terapia ormonale nella donna. Dall'adolescenza alla post-menopausa (in Italian). CIC Edizioni Internazionale: Rome.

SINGH S, SHEPPARD MC AND LANGMAN MJ. (1993). Sex differences in the incidence of colorectal cancer: an exploration of oestrogen and progesterone receptors. *Gut*, **34**, 611–615.

WEISS NS, DALING JR AND CHOW WH. (1981). Incidence of cancer of the large bowel in women in relation to reproductive and hormonal factors. *J. Natl Cancer Inst.*, **67**, 57–60.

WHO (WORLD HEALTH ORGANIZATION). (1992). *Cancer Incidence in Five Continents*. Vol.VI. IARC Scientific Publications no. 120. WHO: Geneva.

WU AH, PAGANINI-HILL A, ROSS RK AND HENDERSON BE. (1987). Alcohol, physical activity and other risk factors for colorectal cancer: a prospective study. *Br. J. Cancer*, **55**, 687–694.

WU-WILLIAMS AH, LEE M, WHITTEMORE AS, GALLAGHER RP, DENG-AO J, LUN Z, XIANGHUI W, KUN C, JUNG D, TEH C-Z, CHENGDE L, YAO XJ, PAFFENBERG RS AND HENDERSON BE. (1991). Reproductive factors and colorectal cancer risk among Chinese females. *Cancer Res.*, **51**, 2307–2311.